# Breast and other cancer dormancy as a therapeutic endpoint: speculative recombinant T cell receptor ligand (RTL) adjuvant therapy worth considering?

**DOI:** 10.1186/1471-2407-10-251

**Published:** 2010-06-02

**Authors:** Tibor Bakács, Jitendra N Mehrishi

**Affiliations:** 1 Department of Probability, Alfred Rényi Institute of Mathematics, Hungarian Academy of Sciences, Reáltanoda utca 13-15, H-1053 Budapest, Hungary; 2 University of Cambridge, Cambridge, United Kingdom; 3 The Cambridge Blood Cell, Stem Cells, Spermatozoa and Opioid Research Initiatives, Macfarlane Cl. 13, Impington, Cambridge CB24 9LZ, UK

## Abstract

**Background:**

Most individuals who died of trauma were found to harbour microscopic primary cancers at autopsies. Surgical excision of the primary tumour, unfortunately, seems to disturb tumour dormancy in over half of all metastatic relapses.

**Presentation of the hypothesis:**

A recently developed immune model suggested that the evolutionary pressure driving the creation of a T cell receptor repertoire was primarily the homeostatic surveillance of the genome. The model is based on the homeostatic role of T cells, suggesting that molecular complementarity between the positively selected T cell receptors and the self peptide-presenting major histocompatibility complex molecules establishes and regulates homeostasis, strictly limiting variations of its components. The repertoire is maintained by continuous peripheral stimulation via soluble forms of self-peptide-presenting major histocompatibility complex molecules governed by the law of mass action. The model states that foreign peptides inhibit the complementary interactions between the major histocompatibility complexes and T cell receptors. Since the vast majority of clinically detected cancers present self-peptides the model assumes that tumour cells are, paradoxically, under homeostatic T cell control.

The novelty of our hypothesis therefore is that resection of the primary tumour mass is perceived as loss of 'normal' tissue cells. Consequently, T cells striving to reconstitute homeostasis *stimulate *rather than inhibit the growth of dormant tumour cells and avascular micrometastases. Here we suggest that such kick-start growths could be prevented by a recombinant T cell receptor ligand therapy that modifies T cell behaviour through a *partial *activation mechanism.

**Testing the hypothesis:**

The homeostatic T cell regulation of tumours can be tested in a tri-transgenic mice model engineered to express potent oncogenes in a doxycycline-dependent manner. We suggest seeding dissociated, untransformed mammary cells from doxycycline naïve mice into the lungs of two mice groups: one carries mammary tumours, the other does not. Both recipient groups to be fed doxycycline in order to activate the oncogenes of the untransformed mammary cells in the lungs, where solitary nodules are expected to develop 6 weeks after injection. We expect that lung metastasis development will be stimulated following resection of the primary tumour mass compared to the tumour-free mice. A recombinant T cell receptor ligand therapy, starting at least one day before resection and continuing during the entire experimental period, would be able to prevent the stimulating effect of surgery.

**Implications of the hypothesis:**

Recombinant T cell receptor ligand therapy of diagnosed cancer would keep all metastatic deposits microscopic for as long as the therapy is continued without limit and could be pursued as one method of cancer control. Improving the outcome of therapy by preventing the development of metastases is perhaps achievable more readily than curing patients with overt metastases.

## Background

Two out of three humans never develop cancer [1]. Nevertheless, most individuals, with no apparent pathology, but who died of trauma, were found to harbour microscopic primary cancers revealed at autopsies [2]. This phenomenon is related to the so-called tumour dormancy, a reference to latent cancer cells. It has been defined as 'the disease free period' between clinical 'cure' of the primary cancer and its subsequent local or distant recurrence/metastasis. Tumour dormancy is a reversible process [3].

Breast cancer, for example, may recur as long as 50 years after surgery [4]. One third of patients, 7 to 22 years after mastectomy and without any evidence of disease, had circulating tumour cells (CTCs). Since the half-life of these CTCs is probably 1 to 3 hours, a precise balance between replication of tumour cells and cell death seems very likely. It has been suggested that the dying CTCs require getting replenished every few hours by replicating tumour cells somewhere in the tissues [5]. Presumably, in a major population of clinically cured patients, 'dormant' breast cancer may be thought of as a chronic disease, which is kept in check by the patients' own physiological mechanisms [6].

Such findings may be explained by the so-called *tumour homeostasis *paradigm [7], which considers tumours as an organ-like structure assuming a dynamic and reciprocal relationship between genetically damaged cells and their microenvironment. The original *seed and soil *hypothesis [8] was refined recognizing that metastases of solid tumours require collaborative interactions between malignant cells and a diverse assortment of "activated" stromal cells at both primary and secondary tumour locations [9].

In the above context, century old observations that surgery of malignant tumours may be the reason for enhancement of growth of metastases with fatal outcome perhaps make sense [7,10-12]. In fact, such experiences often prevented surgeons from touching the tumour if it was not absolutely necessary. Particularly, data in fourteen separate databases from different countries indicated the existence of two peaks in relapse frequency for distal plus local relapses among early stage breast cancer patients, who were untreated with adjuvant therapy (see for references in [12]). As suggested earlier [13], this complex relapse pattern cannot be explained simply by unrestrained continuous cellular growth. Rather the dominant mode of relapse in early-stage breast cancer could be the iatrogenic interference with and most unfortunately terminating dormancy at the time of surgery, precipitating accelerated relapses [14]. In fact, over half of all metastatic relapses seem to be induced by such iatrogenic events [12]. Paradoxically, it has also been shown that a primary tumour may inhibit the growth of metastases in the liver [15].

Compounding the above problem that despite complete resection of the tumour with tumour free margins in all patients, during the following 3 to 4 days, in 85% of treated patients epithelial cells started increasing up to a thousand fold. After resection of tumours, even if they are small and resection is complete, cells can remain in the circulation over long periods of time [16].

Since dormant tumour cells are highly refractory to chemotherapy, leading to uncertainty in the prognosis for patients already treated for primary cancer, a NIH workshop envisioned tumour dormancy as a therapeutic endpoint [17]. Such an objective however requires chronic therapy.

Importantly, the surgical reactivation of metastasis is just one of many other possible theories explaining surgery-induced immune suppression, which increases with larger incisions [18]. In fact, concerns of tumour dissemination related to tumour manipulation led to the explosion of the minimally invasive surgery in the 1990s. Almost every procedure traditionally performed via laparotomy has been performed successfully by laparoscopic methods, including pancreaticoduodenectomy for cancer [19].

## Presentation of the hypothesis

If the majority of humans harbour microscopic cancer and the switch of these tumours to the angiogenic phenotype is so infrequent, then this "Achilles' heel" of cancer progression could be exploited to prevent the growth of many harmless early lesions to the few lethal tumours, currently detectable and treatable [20]. In fact, recently in this journal, Retsky et al. [21] suggested such a therapy for early stage breast cancer. To prevent micrometastatic angiogenesis resulting from surgery or at any time later, they suggest employing the primary antiangiogenic Endostatin starting at least one day before surgery and continuing indefinitely since no acquired resistance develops.

**Figure 1:**
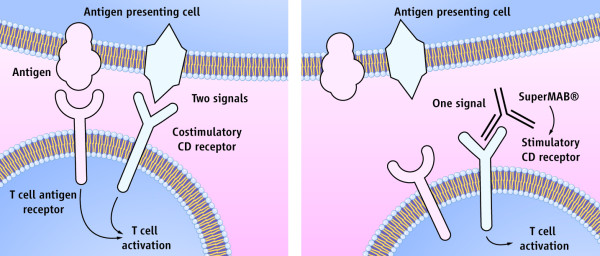
**Strong medicine. The human T cell, a multitasking agent in the immune system, is normally activated only when two receptors are stimulated (left)**. But the "superagonist" used in a London clinical trial can activate T cells by stimulating a single receptor (right). Credit: (illustration) C. Bickel/*Science*. Reproduced with permission from Marshall E. Drug trials. Violent reaction to monoclonal antibody therapy remains a mystery. Science 2006;311(5768):1688-9.

**Figure 2:**
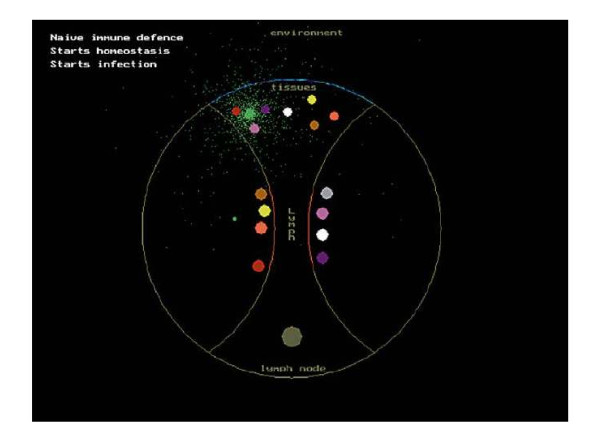
**A single frame is presented from the video clip of the Homeostatic Role of T cells model**. Reproduced with permission of S. Karger AG, Basel from Bakacs T, Mehrishi JN, Szabados T, Varga L, Szabo M, Tusnady G. T Cells Survey the Stability of the Self: A Testable Hypothesis on the Homeostatic Role of TCR-MHC Interactions. Int Arch Allergy Immunol 2007;144:171-82.

Persistent pro-tumour immune responses (inflammation), now generally accepted as potentiating primary tumour development, are being recognized as mediators of cancer metastasis [22]. Therefore, inhibition of metastases at secondary sites by fostering maintenance of immune and tissue homeostasis offers promising approach for cancer therapy when dormant malignant disease might be suspected to occur. In order to prevent the kick-start growth of micrometastases by surgical resection of the primary tumour, we propose a predictably feasible new 'physiological' therapy approach, which is based on the homeostatic role of T cells model [23]. We believe that crucially the virtue of our hypothesis is that the evolutionary pressure driving the creation of a T cell receptor (TCR) repertoire was primarily the homeostatic surveillance of the genome. The TCR repertoire appears to provide a complementary image of the host proteins expressed such that it facilitates the formation of a homeostatic coupled system that restricts variation of T cells and host cells. The TCR repertoire is maintained by continuous peripheral stimulation via soluble forms of self-peptide-presenting major histocompatibility complex (MHC) molecules governed by the law of mass action [24] [see Fig. 1, Fig.2 and video clip in additional file 1 and also in [23,25].

The homeostatic T cell model states that only foreign peptides inhibit the complementary TCR-MHC interactions. Since the vast majority of clinically detected cancers present self-peptides - because they lack tumour associated antigens - the homeostatic T cell model assumes that tumour cells are, paradoxically, controlled by homeostatic T cells. The novelty of our hypothesis therefore is that resection of the primary tumour mass is perceived as loss of 'normal' tissue cells. Consequently, T cells striving to reconstitute homeostasis *stimulate *rather than inhibit the growth of dormant tumour cells and avascular micrometastases. An example that may enlighten our hypothesis is liver resection for metastatic (colorectal carcinoma) tumours, often followed by a significant incidence of tumour recurrence. This might indicate that factors involved in liver regeneration may also stimulate the growth of 'sleeping' tumours and the reactivation of dormant micrometastases [26].

We suggest therefore that such homeostatic T cell stimulation could be prevented by a recombinant TCR ligand ("RTL"), which is composed of a naturally processed peptide product from normal mammary cells covalently ligated to a major histocompatibility complex class I molecule. Appropriate normal (non-tumour-type-specific) peptides may be identified by genomic and proteomic methodologies using cell-displayed peptide libraries carrying intrinsic fluorescent marker allowing for sorting and characterization with quantitative flow cytometry [27].

RTLs - the peptide binding/T cell recognition domains of MHC class II molecules - have emerged as a new class of therapeutic agents with potent clinical efficacy in a diverse set of animal models for multiple sclerosis [28]. The RTL molecule consists of the peptide-binding beta1alpha1 domain of MHC class II covalently ligated to a peptide separating it from the alpha2beta2 domain that may provide additional signalling through CD4 or other cell surface molecules. RTL can bind directly to the TCR and present native antigenic peptides in the context of MHC - i.e. an optimal signal 1 - *without *costimulation from antigen presenting cells (APCs). This way, RTL modifies T cell behaviour through a *partial *activation mechanism that accounts for the inhibitory effects of RTL on encephalitogenic CD4^+ ^T cells. Here we suggest that an RTL therapy would be able to inhibit the homeostatic T cell stimulation of disseminated tumour cells (after resection of the primary tumour) through a partial activation mechanism. It would prolong the natural tendency for dormancy in the early stages of the disease, keep all metastatic deposits microscopic for as long as the therapy would continue without limit and could be pursued as one method of cancer control. Breast cancer is the most obvious target, but this idea could be applied to other cancers as well.

Clearly, an RTL therapy cannot substitute the antigen specific immunotherapeutic strategies aimed at activating the immune system to recognize and destroy tumour cells (e.g. cancer vaccine to stimulate p53-specific T cells using autologous dendritic cells pulsed with various HLA-A*0201 binding wild-type p53 derived peptides used in HLA-A2+ patients with progressive late-stage metastatic breast cancer [29]). Rather the two approaches would probably complement each other. A good place to start such combined efforts is melanoma, which has long been recognized to be immunogenic; furthermore, the clinical facts confirm the metastatic dormancy of melanoma. Definitive proof is available that the immunogenic character of this cancer is associated with dormancy. Melanoma cells persisted in a dormant state in the kidney of a donor for 16 years and upon transplantation to two different but immune-suppressed hosts, they emerged from dormancy [30]. In fact, clinical and experimental data on melanoma indicate that some aspects of melanoma biology imitate traits recently associated with dormancy in other solid cancers [31]. Cutaneous and uveal melanoma, in spite of being of the same origin, differs profoundly in their clinical progression. Between 40 and 50% of uveal melanoma remain undetected for longer than a decade, while less than 5% of cutaneous melanoma show this behaviour. This difference may also have immunologic origin. The eye, the site of uveal melanoma, is rich in immunosuppressive and anti-inflammatory factors, which dampen both innate and adaptive immune responses [31].

## Testing the hypothesis

The protective effect of RTL therapy on the iatrogenic effect of primary tumour resection could be tested in a tri-transgenic mouse model engineered to express potent oncogenes in a doxycycline-dependent mammary-specific manner [32]. Such animals develop diffuse autochthonous tumours only after doxycycline exposure within 3 to 4 weeks. Podsypanina *et al*. [33] demonstrated that in the absence of oncogene activation untransformed mammary cells are capable of travelling to and surviving in the lungs for up to 16 weeks, grow slowly and remain clinically undetectable. Such cells, however, may assume malignant growth upon oncogene activation.

We suggest therefore seeding of dissociated primary mammary cells from doxycycline naïve mice, via the lateral tail veins, in the lungs of two groups of mice: the first group unexposed to doxycycline and the second one with primary mammary tumours developed following doxycycline exposure. Now both recipient mice groups are administered doxycycline to activate the oncogenes of the untransformed mammary cells in the lungs, where solitary nodules are expected to develop 6 weeks after injection [33]. At the start of this feeding animals in both the groups would undergo a sham resection in the first group and resection of the primary tumour in the second group. Based on the prediction of the homeostatic T cell model we would expect that lung metastasis development will be faster in animals from which the primary tumour mass had been removed.

We envisage that an RTL therapy, starting at least one day before surgery and continuing during the entire experimental period, would be able to prevent the growth stimulating effect of surgery.

## Implications of the hypothesis

Jørgensen and Gøtzsche [34] recently reported 52% overdiagnosis of breast cancer in a population offered organised mammography screening - that is, one in three breast cancers is over diagnosed. Others [35-37] judged that screening for cancer, while beneficial, might lead to over diagnoses *and *over treatment, thus perhaps it is best to leave it alone. Sadly, this would mean that 'one in 400 women not screened for any potential treatment for tumour remaining undiagnosed will die within the next 10 years' [38].

Leaving the choice to patients rather than to highly experienced professionals in a matter of almost 'life and death', would place an intolerable burden on those already distressed and looking for sound decisions. Using adjuvant RTL therapy, however, one may not have to choose between the evils of undiscovered primary tumours in unscreened patients and induction of metastases in treated ones - like Odysseus having to choose between two life-threatening evils, Scylla and Charybdis, but managing to avoid both.

## Competing interests

The authors declare that they have no competing interests.

## Authors' contributions

TB conceived of the hypothesis and drafted the manuscript. JNM participated in explanation of how surgery could enhance the growth of metastases and participated in writing the manuscript.

## Pre-publication history

The pre-publication history for this paper can be accessed here:

http://www.biomedcentral.com/1471-2407/10/251/prepub

## Supplementary Material

Additional file 1 **Video clip**. The body is constantly attacked by infections and defended by a collection of antibodies. The infections are represented by 'lightings' that have colours: light or dark blue and silver. The antibody filter has the same colours. The circle is the body containing a tube, which is the lymphoid vessel. The tissue cells (coloured red, yellow, white, brown and purple) are in the central upper part. T cells are located in the lymph node at the edge of the lymphoid vessel. A large APC is located in the lower part. Tissue cell-derived peptides determine TCR specificity. Colours of T cells designate the self pMHCs, which rescued them during positive selection. Complementary self pMHCs and TCRs have identical colours. For the sake of simplicity, in the animation the many different self pMHCs in a single cell are neglected. One T cell recognizes only one self pMHC. The animation shows how the whole system is capable of determining whether a given pMHC is self or non-self. An individual T cell is unable to make such a decision. Together, however, they can because there is a complementary TCR for every self pMHC in the immune system that recognizes an individual peptide fragment. The self pMHCs are presented, one by one, as they flow via the lymph into the lymph node. Eventually, all soluble self pMHCs are captured by complementary TCRs. In this way all tissue cells remain intact. Finally, a viral infection, designated by green colour, enters the body and infects a cell changing its self peptide into a foreign peptide (*f*pMHC). The soluble *f *pMHC molecule (the danger signal in the Homeostatic Role of T cells model) freely crosses the lymphoid tissue because no complementary TCR is present. Eventually, *f*pMHC is captured by an APC, which initiates 2 independent processes. Firstly, the APC activates cytotoxic T cells to locate and eliminate the infection. The T cells travel via the blood vessel into the tissues. In the meantime the virus infects other cells in the body and is also released to the environment represented by small green dots. Secondly, the APC initiates hypermutation in B cells represented by coloured dots at the lower right part of the screen. Eventually, the green colour will appear as a new B cell clone and also become part of the extended immune defence filter. Click here for file
